# Accumulation of Unusual Gangliosides G_Q3_ and G_P3_ in Breast Cancer Cells Expressing the G_D3_ Synthase

**DOI:** 10.3390/molecules17089559

**Published:** 2012-08-10

**Authors:** Agata Steenackers, Jorick Vanbeselaere, Aurélie Cazet, Marie Bobowski, Yoann Rombouts, Florent Colomb, Xuefen Le Bourhis, Yann Guérardel, Philippe Delannoy

**Affiliations:** 1 Structural and Functional Glycobiology Unit, UMR CNRS 8576, University of Sciences and Technologies of Lille, 59655 Villeneuve d’Ascq, France; Email: agata.steenackers@hotmail.fr (A.S.); jorick.vanbeselaere@gmail.com (J.V.); aurelie_cazet@yahoo.fr (A.C.); marie.bobowski@hotmail.fr (M.B.); yoann.rombouts@gmail.com (Y.R.); florent.colomb@ed.univ-lille1.fr (F.C.); yann.guerardel@univ-lille1.fr (Y.G.); 2 INSERM U908, University of Sciences and Technologies of Lille, 59655 Villeneuve d’Ascq, France; Email: xuefen.lebourhis@univ-lille1.fr

**Keywords:** gangliosides, G_D3_ synthase, MCF-7, unusual gangliosides

## Abstract

Glycosphingolipids from the ganglio-series are usually classified in four series according to the presence of 0 to 3 sialic acid residues linked to lactosylceramide. The transfer of sialic acid is catalyzed in the Golgi apparatus by specific sialyltransferases that show high specificity toward glycolipid substrates. ST8Sia I (EC 2.4.99.8, SAT-II, SIAT 8a) is the key enzyme controlling the biosynthesis of b- and c-series gangliosides. ST8Sia I is expressed at early developmental stages whereas in adult human tissues, ST8Sia I transcripts are essentially detected in brain. ST8Sia I together with b- and c-series gangliosides are also over-expressed in neuroectoderm-derived malignant tumors such as melanoma, glioblastoma, neuroblastoma and in estrogen receptor (ER) negative breast cancer, where they play a role in cell proliferation, migration, adhesion and angiogenesis. We have stably expressed ST8Sia I in MCF-7 breast cancer cells and analyzed the glycosphingolipid composition of wild type (WT) and GD3S+ clones. As shown by mass spectrometry, MCF-7 expressed a complex pattern of neutral and sialylated glycosphingolipids from globo- and ganglio-series. WT MCF-7 cells exhibited classical monosialylated gangliosides including G_M3_, G_M2_, and G_M1a_. In parallel, the expression of ST8Sia I in MCF-7 GD3S+ clones resulted in a dramatic change in ganglioside composition, with the expression of b- and c-series gangliosides as well as unusual tetra- and pentasialylated lactosylceramide derivatives G_Q3_ (II^3^Neu5Ac_4_-Gg_2_Cer) and G_P3_ (II^3^Neu5Ac_5_-Gg_2_Cer). This indicates that ST8Sia I is able to act as an oligosialyltransferase in a cellular context.

## Abbreviations:

BSA: Bovine Serum Albumin
Cer: ceramide
DMB: 1,2-diamino-4,5-methylenedioxybenzene
DMEM: Dulbecco’s Modified Eagle’s Medium
EDTA: Ethylenediaminetetraacetic Acid
DMSO: Dimethyl Sulfoxide
FBS: Fetal Bovine Serum
FITC: Fluorescein Isothiocyanate
FL-HPLC: Fluorescence Detection High Performance Liquid Chromatography
GD3S: G_D3_ synthase
GSL: glycosphingolipid
HPRT: Hypoxanthine PhosphoRibosylTransferase
HRP: horseradish peroxidase
LacCer: Lactosylceramide
mAb: monoclonal Antibody
MALDI-TOF: matrix assisted laser desorption-ionization time-of-flight
MS: Mass Spectrometry
PBS: Phosphate Buffered Saline
QPCR: Quantitative real-time Polymerase Chain Reaction
SDS-PAGE: Sodium Dodecyl Sulfate Polyacrylamide Gel Electrophoresis
ST3Gal V: G_M3_ synthase
ST8Sia I: G_D3_ synthase
ST8Sia V: G_T3_ synthase; WT: Wild Type

## 1. Introduction

**Figure 1:**
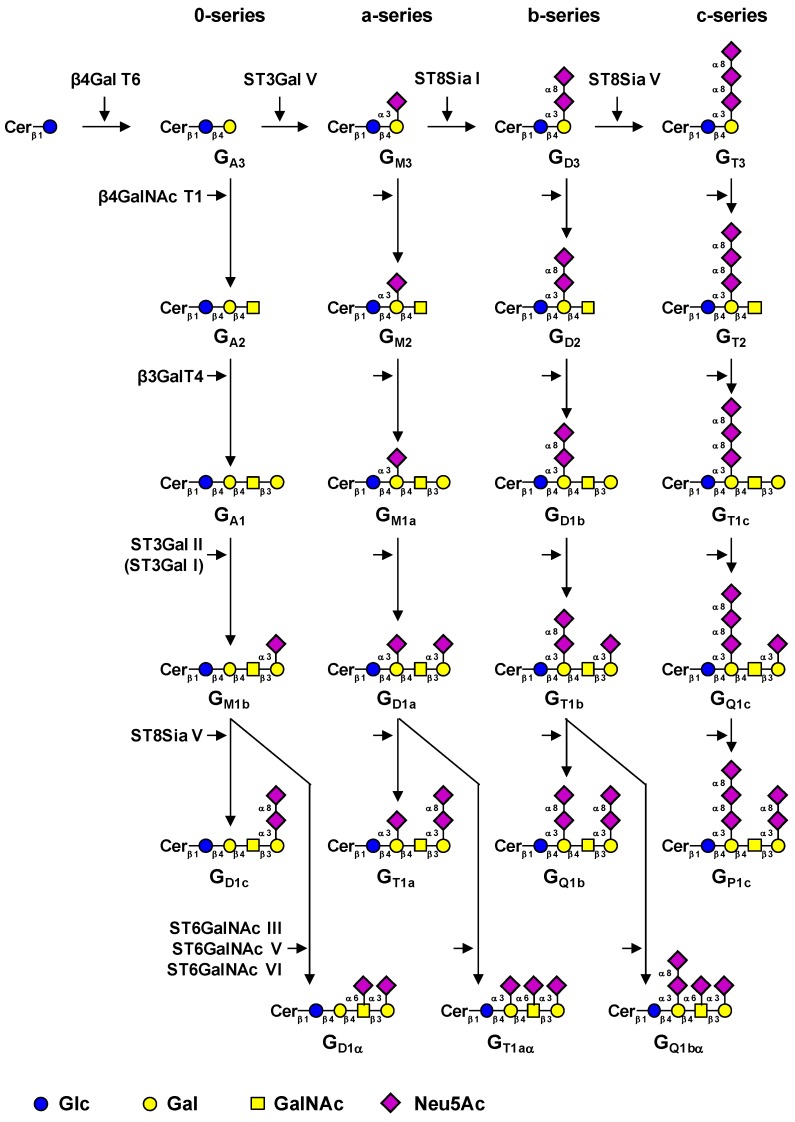
Biosynthesis pathway for gangliosides. The code names of gangliosides are according to Svennerholm [1]. Cer: ceramide.

Glycosphingolipids (GSL) from the ganglio-series are classified in four series according to the presence of 0 to 3 sialic acid residues linked to lactosylceramide (Galβ1-4Glc-Cer, LacCer) [1]. The transfer of sialic acid to LacCer is catalyzed in the Golgi apparatus by specific sialyltransferases (namely ST3Gal V, ST8Sia I and ST8Sia V) that show high specificity toward glycolipid substrates [2]. LacCer, G_M3_ (Neu5Acα2-3Galβ1-4Glc-Cer, II^3^Neu5Ac_1_-Gg_2_Cer), G_D3_ (Neu5Acα2-8Neu5Acα2-3Galβ1-4Glc-Cer, II^3^Neu5Ac_2_-Gg_2_Cer) and G_T3_ (Neu5Acα2-8Neu5Acα2-8Neu5Acα2-3Galβ1-4Glc-Cer, II^3^Neu5Ac_3_-Gg_2_Cer) are therefore the precursors for 0-, a-, b- and c-series gangliosides and the biosynthesis of these compounds determine the relative proportion of gangliosides in each series (Figure 1). Elongation of the precursors can then occur by the sequential action of *N*-acetyl-galactosaminyltransferase (β4GalNAc T1), galactosyltransferase (β3Gal T4) and sialyltransferases (ST3Gal I, ST3Gal II and ST8Sia V), α-gangliosides deriving from the action of ST6GalNAc III, V or VI on G_M1b_, G_D1a_ or G_T1b_ (Table 1).

The sialyltransferase ST8Sia I (EC 2.4.99.8, SAT-II, SIAT 8a) is the only enzyme known to catalyze the transfer of a sialic acid residue onto G_M3_ through an α2,8-linkage to synthesize G_D3_. ST8Sia I and G_D3_ are expressed in fetal tissues at an early developmental stage [3,4] where they play a key role in cell-cell interaction, cell differentiation and proliferation [5], whereas in adult human tissues, ST8Sia I is essentially detected in the brain [6]. ST8Sia I and G_D3_ have been also shown to be over-expressed in neuroectoderm-derived malignant tumors such as melanoma, glioblastoma and neuroblastoma, and in estrogen receptor negative breast cancer [7,8,9,10].

**Table 1:** Glycosyltransferases involved in gangliosides biosynthesis. R = G_A3_, G_M3_, G_D3_ or G_T3_.

*Gene*	*Common name*	*Main acceptor(s)*	*Accession #*	*Reference*
*UGCG*	GlcCer synthase	Ceramide	NM_003358	[18]
*B4GALT6*	LacCer synthase	Glucosylceramide	NM_004775	[19,20]
*ST3GAL5*	G_M3_ synthase	Lactosylceramide	NM_003896	[21]
*ST8SIA1*	G_D3_ synthase	G_M3_, G_D3_	NM_003034.2	[11,12,13]
*ST8SIA5*	G_T3_ synthase	G_D3_, G_M1b_, G_D1a_, G_T1b_	NM_013305	[17]
*B4GALNACT1*	G_M2_/G_D2_ synthase	G_A3_, G_M3_, G_D3_, G_T3_	NM_001478.2	[22,23,24]
*B3GALT4*	G_M1a_/G_D1b_ synthase	G_A2_, G_M2_, G_D2_, G_T2_	NM_003782.3	[23,25]
*ST3GAL1*	ST3Gal I	Galβ1-3GalNAcβ1-4-R	NM_003033	[26]
*ST3GAL2*	ST3Gal II	Galβ1-3GalNAcβ1-4-R	NM_006927	[27]
*ST6GALNAC3*	ST6GalNAc III	Neu5Acα2-3Galβ1-3GalNAcβ1-4-R	NM_152996	[28]
*ST6GALNAC5*	ST6GalNAc V	Neu5Acα2-3Galβ1-3GalNAcβ1-4-R	NM_030965.1	[29]
*ST6GALNAC6*	ST6GalNAc VI	Neu5Acα2-3Galβ1-3GalNAcβ1-4-R	NM_013443.3	[29]

The human ST8Sia I cDNA was simultaneously isolated by expression cloning by three research groups [11,12,13]. The *ST8SIA1* gene is located on chromosome 12, in p12.1-p11.2 and consists of five coding exons spanning over 135 kbp of genomic DNA [14]. ST8Sia I cDNA encodes a 341 amino acid membrane-bound Golgi enzyme with a 12 amino-acid cytoplasmic tail, a transmembrane domain of about 20 residues and a catalytic domain containing the conserved Sialyl motifs involved in substrate binding and transfer [15].

Whereas ST8Sia I mainly sialylates G_M3_, Nakayama and co-workers have underlined its ability to synthesize G_T3_ from G_D3_ [6]. ST8Sia I was also shown to use G_M1b_, G_D1a_ or G_T1b_ as acceptor substrates to synthesize G_D1c_, G_T1a_ or G_Q1b_, respectively, both *in vitro* and *in vivo* [16]. However, the α2,8-sialyltransferase ST8Sia V is a much better candidate for G_T1a_/G_Q1b_ synthase activity [17] and no ST8Sia V activity was detected toward G_M3_. Consequently, ST8Sia I is considered as the only G_D3_ synthase (GD3S) that controls the biosynthesis of gangliosides from the b- and c-series.

By stable transfection of the full-length cDNA of human G_D3_ synthase, we have isolated cellular clones deriving from MCF-7 breast cancer cells that constitutively express GD3S together with b- and c-series gangliosides. Here, we show by mass spectrometry and HPLC analysis that clones that express a high level of GD3S also accumulate unusual tetra- and pentasialylated derivatives of LacCer, G_Q3_ (II^3^Neu5Ac_4_-Gg_2_Cer) and G_P3_ (II^3^Neu5Ac_5_-Gg_2_Cer). 

## 2. Results and Discussion

### 2.1. Analysis of ST8Sia I Expression by QPCR in Control and GD3S+ MCF-7 Clones

**Figure 2:**
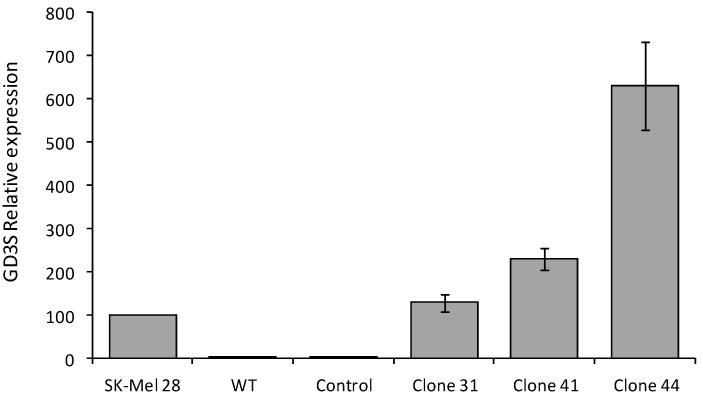
QPCR analysis of GD3S expression in control and GD3S+ MCF-7 clones. Quantification of GD3S expression was performed by the method described by Livak and Schmittgen [32] and normalized to HPRT. The expression of GD3S in MCF-7 cells was relative to SK-Mel 28, which was regarded as 100%.

MCF-7 cells were transfected with the pcDNA3-GD3S expression vector containing the full-length cDNA of human GD3S or the empty pcDNA3 vector as control. Transfected cells were cultured 21 days in the presence of 1 mg/mL G418. Individual G418-resistant colonies were isolated by limiting dilution cloning. Forty-four clones were obtained and analyzed for the expression of GD3S. As previously shown [30], QPCR analysis of GD3S expression (Figure 2) indicates that GD3S mRNA is express at a very low level in wild-type and control (empty vector transfected) MCF-7 cells compared to SK-Mel 28 melanoma cells used as positive control [31]. Within the forty-four analyzed clones, three GD3S+ clones (clone #31, #41 and #44) were selected according to the high expression of GD3S compared to SK-Mel 28 (1.3-fold, 2.3-fold and 6.3-fold, respectively) (Figure 2).

### 2.2. Flow Cytometry Analysis of Gangliosides Expression in MCF-7 GD3S+ Clones

**Figure 3:**
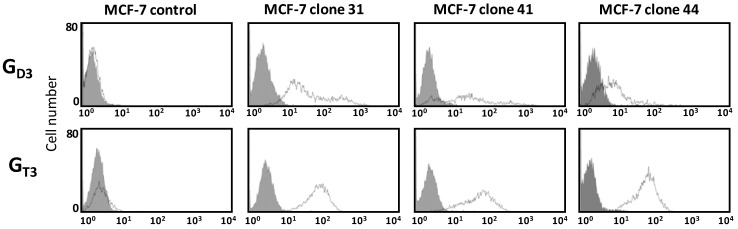
Flow cytometry analysis of gangliosides in pcDNA3-GD3S transfected MCF-7 cells. Immunodetection of G_D3_ and G_T3_ was performed using anti-G_D3_ R24 (dilution 1:100) and anti-G_T3_ A2B5 (dilution 1:10) mAbs. The gray peaks correspond to the negative controls (incubation with secondary antibody alone, anti-mouse IgM or anti-mouse IgG labeled with Alexa 488).

The pattern of gangliosides was monitored in the three selected MCF-7 GD3S+ clones (clone #31, #41, #44) by flow cytometry using anti-G_D3_ R24 and anti-G_T3_ A2B5 mAbs. As shown in Figure 3, the three GD3S+ clones expressed G_D3_ and G_T3_ whereas wild-type and control (empty vector transfected) MCF-7 cells did not expressed complex gangliosides. G_T3_ is expressed at a similar level in the three GD3S+ clones but a decrease of G_D3_ is observed in clone #44 compared to clone #31 and #41 whereas the expression level of GD3S was 4.8-fold or 2.7-fold higher in clone #44 compared to clone #31 and #41, respectively (Figure 2). Control cells showed no change in the ganglioside profile compared with wild-type MCF-7 (data not shown).

### 2.3. MS Analysis of Gangliosides in MCF-7 and GD3S+ Clones

**Figure 4:**
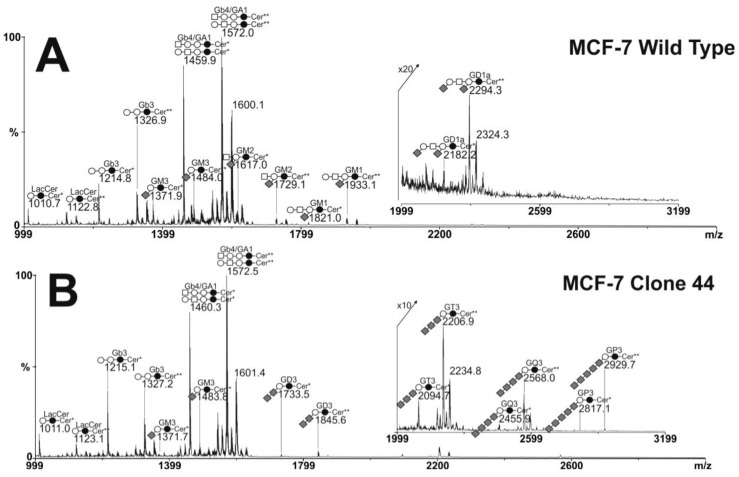
Comparison of MS profiles of permethylated glycosphingolipids purified from MCF-7 WT and GD3S+ clone #44. All GSLs are present as d18:1/C16:0 (Cer*) and d18:1/C24:0 (Cer**) isomers. 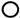, Gal; 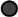, Glc; 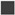, GalNAc; 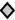, Neu5Ac.

Glycolipids were extracted from cells, purified by reverse phase chromatography and permethylated prior to MS analysis. Mass spectrometry analysis established that glycolipid profiles of MCF-7 WT and GD3S+ clones were characterized by complex patterns of neutral and sialylated glycosphingolipids from globo- and ganglio-series. Profiles of all cell lines were dominated by two signals at *m/z* 1460 and 1572 both identify based on their MALDI-TOF/TOF fragmentation patterns (data not shown) and in agreement with previously published analyses [33] as mixtures of G_b4_ and G_A1_ differing by the nature of their lipid moieties (d18:1-16:0 or d18:1-24:0). Along these two major components, MS and MS/MS analyses permitted us to identified other minor neutral GSLs including LacCer and G_b3_ (Figure 4). The comparison of MS profiles did not show any significant difference in neutral GSLs content between MCF-7 WT and GD3+ clones. On the contrary, the content in sialylated glycolipids varied among the different cell lines. MCF-7 WT cells exhibited monosialylated gangliosides including G_M3_, G_M2_, and G_M1_. The only disialylated GSL observed in MCF-7 WT cells was G_D1_ at *m/z* 2182 and 2194. Its sequence analysis by MALDI-TOF/TOF typified it as G_D1a_, thus lacking disialylated motif (data not shown). GD3+ clones did not show GSLs from the G2 and G1 families but synthesized instead a family of unusual highly sialylated lactosylceramide derivatives substituted by up to 5 Neu5Ac residues tentatively identified as G_D3_, G_T3_, G_Q3_ and G_P3_. The structure of these four compounds was confirmed by MALDI-TOF/TOF sequencing. 

The Glycolipid profile of SK-Mel 28 cells was also analyzed in respect to the presence of polysialylated lactosylceramide derivatives. Contrarily to MCF-7 WT cells, GSLs extracted from SK-Mel 28 cells did not contain globo-series but were dominated by ganglio-series, which induces much higher overall sialic acid content (data not shown). In particular, disialylated G_D3_ appears to be the major component along with monosialylated G_M3_ and trisialylated G_T3_. However, the tetra- and pentasialylated gangliosides synthesized by MCF-7 GD3+ clones were not detected in SK-Mel 28.

**Figure 5:**
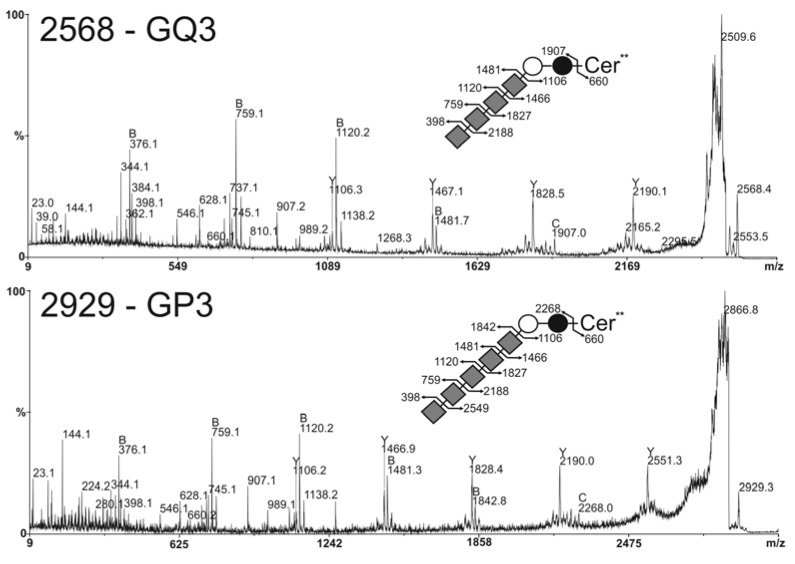
MS/MS sequencing of permethylated glycosphingolipids. Analysis of G_Q3_ at *m/z* 2568 (**A**) and G_P3_ at *m/z* 2929 (**B**) with ceramide moieties d18:1/C24:0 (Cer**). All fragments are observed as [M+Na]^+^ adducts except those at *m/z* at 376 and 660 observed as [M+H]^+^ adducts. Fragment ions were annotated according to nomenclature of Domon and Costello [34]. 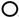, Gal; 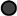, Glc; 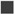, GalNAc; 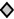, Neu5Ac.

We illustrate the sequence analyses of unusual G_Q3_ and G_P3_ in Figure 5. These two molecules differing in the presence of a single Neu5Ac residue were structurally related. They shared the fragmentation pattern of a linear stretch of four sialic acid residues in terminal non-reducing position as [M+Na]^+^ B-ions at *m/z* 398, 759, 1120 and 1481 and a linear sequence of Sia_4_Hex_2_Cer at reducing end as [M+Na]^+^ Y-ions at *m/z* 1106, 1466, 1827 and 2188. G_P3_ showed additional B and Y ions at *m/z* 1842 and 2551 typifying a linear Sia_5_ sequence. Altogether, these data established that MCF-7 GD3+ clone #44 synthesizes an unusual family of oligosialylated lactosylceramide derivatives presented from 2 to 5 Neu5Ac residues. The disappearance of G_M2_, G_M1_ and G_D1a_ in MCF-7 GD3+ clone #44 could be explained by the depletion of G_M3_ substrate caused by the over-expression of the GD3S+ that competes with β-4GalNAcT1 for the use of G_M3_ substrate.

### 2.4. Quantification of Polysialylation Associated with Gangliosides by HPLC

**Figure 6:**
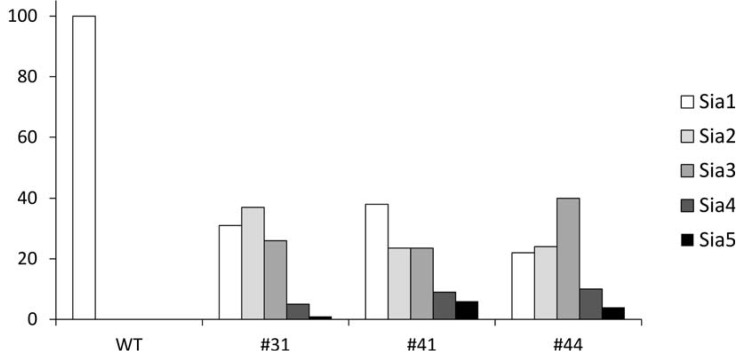
Relative quantification of oligosialylation sequence on GSL of MCF-7 WT GD3S+ clones #31, clone #41 and clone #44 by FL-HPLC after DMB derivation.

Because mass spectrometry does not provide reliable quantitative data, we quantified the extent of oligosialylation modifications in GSLs induced by the overexpression of G_D3_ in MCF-7 by screening all three GD3S+ clones (#31, #41 and #44) (Figure 6). To do that, oligosialylated motifs were released from purified GSLs by mild hydrolysis according to optimized procedures [35] and labeled by DMB before separation and quantification by FL-HPLC [36]. The analysis of relative quantifications of sialic acid chains in GSLs of all MCF-7 GD3S+ clones compared to MCF-7 WT demonstrated a sharp increase of sialylation oligomerization degree, up to five residues, in accordance with mass spectrometry analysis. In MCF-7 WT, no oligosialylation could be observed, in accordance with the sole identification of G_D1a_ by MS. In contrast, all GD3S+ clones contained about 30% of Sia_2_ and Sia_3_ motifs, up to 10% of Sia_4_ and up to 5% of Sia_5_. Although clones presented similar GSL oligosialylation profiles, small scale quantitative differences could be observed between the three clones, with a prevalence of higher DP values for clone #44 compared to clones #31 and #41.

## 3. Experimental Section

### 3.1. Antibodies and Reagents

Anti-G_D3_ R24 mAb was purchased from Abcam (Cambridge, UK) and anti-G_T3_ A2B5 mAb was kindly provided by Pr. Jacques Portoukalian (Depart. of Transplantation and Clinical Immunology, Claude Bernard University and Edouard Herriot Hospital, Lyon, France). FITC-conjugated sheep anti-mouse IgG was from GE Healthcare (Templemars, France). FITC-conjugated anti-mouse IgM was purchased from Molecular Probes (Invitrogen, Carlsbad, CA, USA).

### 3.2. Cell Culture

The breast cancer cell line MCF-7 and the melanoma cell line SK-Mel 28 were obtained from the American Type Cell Culture Collection. Cell culture reagents were purchased from Lonza (Levallois-Perret, France). Cells were routinely grown in monolayer and maintained at 37 °C in an atmosphere of 5% CO_2_, in Dulbecco’s modified Eagle’s medium (DMEM) supplemented with 10% fetal bovine serum (FBS), 2 mM L-glutamine, and 100 units/mL penicillin-streptomycin. GD3S positive (GD3S+) MCF-7 clones were obtained by stable transfection of the pcDNA3-GD3S expression vector encoding the full-length human G_D3_ synthase [37] as previously described [33]. Individual resistant colonies were isolated by limit dilution. Three positive clones (#31, #41 and #44), expressing different levels of GD3S were used for further study. Control cells (empty vector transfected) and GD3S+ clones were cultured in the presence of 1 mg/mL G418 (Invitrogen, Cergy-Pontoise, France).

### 3.3. Quantitative Real-Time-PCR (QPCR) Analysis of G_D3_ Synthase

Total RNA was extracted using the Nucleospin RNA II kit (Macherey Nagel, Hoerdt, France), quantified using a NanoDrop spectrophotometer (Thermo Scientifics, Wilmington, USA) and the purity of the preparation was checked by ratio of the absorbance at 260 and 280 nm. The cDNA was synthesized using 2 µg of RNA (GE Healthcare). PCR primers for GD3S and Hypoxanthine PhosphoRibosylTransferase (HPRT) were previously described [30,38] and synthesized by Eurogentec (Seraing, Belgium). PCR reactions (25 µL) were performed using 2X SYBR^®^ Green Universal QPCR Master Mix (Stratagene, Amsterdam, The Netherlands), with 2 µL of cDNA solution and 300 nM final concentration of each primer. PCR conditions were as follows: 95 °C for 30 s, 51 °C for 45 s, 72 °C for 30 s (40 cycles). Assays were performed in triplicate and GD3S transcript expression level was normalized to HPRT using the 2^−^^ΔΔCt^ method described by Livak and Schmittgen [32]. Serial dilutions of the appropriate positive control cDNA sample were used to create standard curves for relative quantification and negative control reactions were performed by replacing cDNA templates by sterile water.

### 3.4. Analysis of Cell Surface Ganglioside by Flow Cytometry

Cells were washed in cold PBS and detached by EDTA 2 mM. Cells were incubated at 4 °C during 1 h with anti-G_D3_ R24 (1:100) and anti-G_T3_ A2B5 (1:10), diluted in phosphate buffered saline (PBS) containing 0.5% bovine serum albumin (PBS-BSA) (Sigma-Aldrich). After washing with PBS-BSA, cells were incubated on ice during 1 h with Alexa Fluor 488 anti-IgG or anti-IgM (1:500). After two washes in PBS-BSA, cells were analyzed by flow cytometry (FACScalibur, Becton Dickinson). Control experiments were performed using secondary antibody alone. 

### 3.5. Extraction and Preparation of Glycolipids

Twenty dishes (10 cm diameter) of cultured cells were washed twice with ice-cold PBS and cells were scraped and sonicated on ice in 200 µL of water. The resulting material was dried under vacuum and sequentially extracted by CHCl_3_/CH_3_OH (2:1, v/v), CHCl_3_/CH_3_OH (1:1, v/v) and CHCl_3_/CH_3_OH/H_2_O (1:2:0.8, v/v/v). Supernatants were pooled, dried and subjected to a mild saponification in 0.1 M NaOH in CHCl_3_/CH_3_OH (1:1) at 37 °C for 2 h and then evaporated to dryness [39]. Samples were reconstituted in CH_3_OH/H_2_O (1:1, v/v) and applied to a reverse phase C_18_cartridge (Waters, Milford, MA, USA) equilibrated in the same solvent. After washing with CH_3_OH/H_2_O (1:1, v/v), GSLs were eluted by CH_3_OH, CHCl_3_/CH_3_OH (1:1, v/v) and CHCl_3_/CH_3_OH (2:1, v/v).

### 3.6. Mass Spectrometry Analysis of GSL

Prior to mass spectrometry analysis, GSL were permethylated according to Ciucanu and Kerek [40]. Briefly, compounds were incubated 2 h in a suspension of 200 mg/mL NaOH in dry DMSO (300 µL) and CH_3_I (200 µL). The methylated derivatives were extracted in CHCl_3_ and washed several times with water. The reagents were evaporated and the sample was dissolved in CHCl_3_ in the appropriate dilution. MALDI-MS and MS/MS analyses of permethylated GSL were performed on 4800 Proteomics Analyzer (Applied Biosystems, Framingham, MA, USA) mass spectrometer, operated in the reflectron mode. For MS acquisition, 5 µL of diluted permethylated samples in CHCl_3_ were mixed with 5 µL of 2,5-dihydroxybenzoic acid matrix solution (10 mg/mL dissolved in CHCl_3_/CH_3_OH (1:1, v/v)). The mixtures (2 µL) were then spotted on the target plate and air dried. MS survey data comprises a total of 50 sub-spectra of 1500 laser shots. Peaks observed in the MS spectra were selected for further MS/MS. CID MS/MS data comprises a total of 100 sub-spectra of 3000 laser shots. Two or more spectra can be combined post-acquisition with mass tolerance set at 0.1 Da to improve S/N ratio. The potential difference between the source acceleration voltage and the collision cell was set to 1 kV and argon was used as collision gas.

### 3.7. Analysis of Oligo-Sialylated Sequences by HPLC

In order to minimize internal fragmentation of polysialylated sequences, sialylated glycan samples were directly coupled to 1,2-diamino-4,5-methylenedioxybenzene (DMB) without prior mild hydrolysis [35]. Samples were incubated for 2.5 h at 50 °C in 50 μL of a DMB reagent solution (2.7 mM DMB, 9 mM sodium hydrosulfite, and 0.5 mM β-mercaptoethanol in 20 mM TFA). 10 μL of 1 M NaOH was then added and the reaction mixtures further incubated in the dark at room temperature for 1 h. Samples were stored at 4 °C before analysis. DMB-derivatized sialic acid oligomers were separated on a HPLC apparatus fitted with a CarboPac PA-100 column (Dionex). CarboPac column was eluted at 1 mL/min with a concentration gradient of 2 to 32% of 1 M NaNO_3_ in water. Elution was monitored by an on line fluorescence detector set at wavelengths of 373 nm for excitation and 448 nm for emission. 

## 4. Conclusions

ST8Sia I is the only sialyltransferase able to transfer a sialic acid residue onto G_M3_ to synthesize G_D3_ and is therefore considered as the G_D3_ synthase. The animal ST8Sia family can be divided in three groups according to the capacity to carry out poly-, oligo- and mono-α2,8-sialylation and phylogenic analyses have clearly associated ST8Sia I to the group of mono-α2,8-sialyltransferase [41]. However, by expression cloning of the human G_T3_ synthase, Nakayama and co-workers have underlined the ability of ST8Sia I to synthesize G_T3_ from G_D3_ [6]. Here, we show, for the first time, that ST8Sia I is able to synthesize unusual highly sialylated lactosylceramide derivatives substituted by up to 5 Neu5Ac residues and identified as G_Q3_ (II^3^Neu5Ac_4_-Gg_2_Cer) and G_P3_ (II^3^Neu5Ac_5_-Gg_2_Cer), showing that this enzyme can act as an oligosialyltransferase. 

In humans, the *ST8SIA1* gene is located on chromosome 12, in p12.1-p11.2 and consists of five coding exons spanning over 135 kbp of genomic DNA [14]. Two initiation codons on the first exon lead to two protein isoforms of 356 or 341 amino acids that differ in their N-terminal part. However, the relative capacity of each isoform to transfer more than two sialic acid residues has not been evaluated. By *in vitro* sialyltransferase assay, a recombinant soluble form of the human G_D3_ synthase was shown to synthesize, after a long period of incubation, higher polysialogangliosides, which presumably have more than three sialic acid residues but these compounds were not characterized [6].

From a general point of view, the occurrence of oligosialylation associated with glycolipids has been rarely reported so far. One exception is the recent identification of polysialogangliosides containing α2,8-linked polyNeu5Ac with DPs ranging from 2 to at least 16 in sea urchin sperm head [42]. However, to our knowledge, tetra- and pentasialylated lactosylceramide derivatives have never been described in human tissues and cells. Although SK-Mel 28 cells that constitutively express high levels of GD3S, synthesize high quantities of G_D3_ and G_T3_, we were not able to detect these unusual tetra- and pentasialylated gangliosides in this cell line. Indeed, in our cellular model, these unusual structures were obtained after transfection of GD3S cDNA and may not exist in natural conditions, and the depletion of G_M3_ in GD3S+ MCF-7 clones could explain that GD3S used other gangliosides, such as G_T3_ or G_Q3_, as acceptor substrates. Nevertheless, one may thus expect that revisiting the structure of human gangliosides with recent high sensitivity mass spectrometry techniques will uncover oligosialylated glycolipids in cancerous cell lines and in normal or pathological tissues.
